# Insulin Protects Cortical Neurons Against Glutamate Excitotoxicity

**DOI:** 10.3389/fnins.2019.01027

**Published:** 2019-09-24

**Authors:** Irina Krasil’nikova, Alexander Surin, Elena Sorokina, Andrei Fisenko, Dmitry Boyarkin, Maxim Balyasin, Anna Demchenko, Igor Pomytkin, Vsevolod Pinelis

**Affiliations:** ^1^National Medical Research Center for Children’s Health, Moscow, Russia; ^2^Institute of General Pathology and Pathophysiology, Russian Academy of Sciences, Moscow, Russia; ^3^Department of Advanced Cell Technologies, Institute of Regenerative Medicine, I.M. Sechenov First Moscow State Medical University, Moscow, Russia; ^4^Scientific Center for Biomedical Technologies, Federal Medical and Biological Agency, Svetlye Gory, Moscow, Russia

**Keywords:** insulin, glutamate excitotoxicity, cortical neuron, delayed calcium deregulation (DCD), mitochondrial depolarization, reactive oxygen species (ROS), BDNF

## Abstract

Glutamate excitotoxicity is implicated in the pathogenesis of numerous diseases, such as stroke, traumatic brain injury, and Alzheimer’s disease, for which insulin resistance is a concomitant condition, and intranasal insulin treatment is believed to be a promising therapy. Excitotoxicity is initiated primarily by the sustained stimulation of ionotropic glutamate receptors and leads to a rise in intracellular Ca^2+^ ([Ca^2+^]_*i*_), followed by a cascade of intracellular events, such as delayed calcium deregulation (DCD), mitochondrial depolarization, adenosine triphosphate (ATP) depletion that collectively end in cell death. Therefore, cross-talk between insulin and glutamate signaling in excitotoxicity is of particular interest for research. In the present study, we investigated the effects of short-term insulin exposure on the dynamics of [Ca^2+^]_*i*_ and mitochondrial potential in cultured rat cortical neurons during glutamate excitotoxicity. We found that insulin ameliorated the glutamate-evoked rise of [Ca^2+^]_*i*_ and prevented the onset of DCD, the postulated point-of-no-return in excitotoxicity. Additionally, insulin significantly improved the glutamate-induced drop in mitochondrial potential, ATP depletion, and depletion of brain-derived neurotrophic factor (BDNF), which is a critical neuroprotector in excitotoxicity. Also, insulin improved oxygen consumption rates, maximal respiration, and spare respiratory capacity in neurons exposed to glutamate, as well as the viability of cells in the MTT assay. In conclusion, the short-term insulin exposure in our experiments was evidently a protective treatment against excitotoxicity, in a sharp contrast to chronic insulin exposure causal to neuronal insulin resistance, the adverse factor in excitotoxicity.

## Introduction

L-Glutamate (glutamate) is the major excitatory neurotransmitter in the central nervous system (CNS) that is involved in most normal brain function, such as cognition, memory, and learning, as well as a specific pathophysiological process called excitotoxicity. The glutamate excitotoxicity was first identified in 1957, when partial necrosis of the mouse retina was observed after parenteral administration of glutamate (Lucas and Newhouse, 1957). Subsequently, glutamate excitotoxicity has been implicated in neuronal death during cerebral ischemia, traumatic brain injuries, and neurodegenerative diseases. The glutamate-induced activation of the ionotropic N-methyl-D-aspartate (NMDA) receptor, followed by a Ca^2+^ influx, is generally considered to be central to the development of excitotoxicity (Nicholls and Budd, 2000; Khodorov, 2004; Nicholls, 2004; Zhou and Sheng, 2013; Lai et al., 2014; Surin et al., 2014b). Experiments with primary neuronal cultures have shown that glutamate exposure causes an initial rapid increase in the intracellular free Ca^2+^ concentration ([Ca^2+^]_*i*_), followed by a larger secondary [Ca^2+^]_*i*_ increase, simultaneous to a decrease in the mitochondrial inner membrane potential (ΔΨ_*m*_) (Tymianski et al., 1993; Kiedrowski and Costa, 1995; Nicholls and Budd, 1998, 2000; Khodorov, 2004; Brittain et al., 2012). The irreversible larger secondary, [Ca^2+^]_*i*_ increase may occur with a delay of minutes to two hours (lag period) and has been called the “delayed calcium overload” (Tymianski et al., 1993) or “delayed calcium deregulation” (DCD) (Nicholls and Budd, 2000). DCD is postulated to be a point-of-no-return in excitotoxicity; in other words, events occurring downstream of DCD onset are considered to influence the timing of cell death without altering its inevitability (Nicholls, 2004). DCD-like reversible secondary calcium elevations (RCE) in certain neurons may precede the irreversible DCD that occurs upon extended glutamate exposure (Schinder et al., 1996; Pivovarova et al., 2008; Gerencser et al., 2009). DCD and components of glutamate excitotoxicity, such as mitochondrial depolarization, opening mitochondrial permeability transition pores, increasing production of reactive oxygen species (ROS) and nitric oxide, and the activation of phospholipases and proteases, collectively lead to neuronal death (Gerencser et al., 2009; Brustovetsky et al., 2010; Wang and Qin, 2010).

Glutamate excitotoxicity has been observed in numerous diseases, such as ischemic stroke, traumatic brain injury (Zhou and Sheng, 2013), and Alzheimer’s disease (Mota et al., 2014), for which insulin resistance has been shown to be a risk factor or a symptom (Rundek et al., 2010; Talbot et al., 2012), and intranasal insulin has been shown to be a promising treatment (Craft et al., 2012; Lioutas and Novak, 2016; Brabazon et al., 2017). Therefore, a particular interest has been dedicated to the cross-talk between insulin and NMDA receptor signaling in the brain in health and disease. It has been reported that insulin receptors and NMDA receptors are both present in synapses, as a component of postsynaptic density, as well as outside of synaptic terminals (Abbott et al., 1999). Insulin enhances NMDA-mediated glutamatergic neurotransmission. Insulin potentiates NMDA receptor currents in a dose-, time-, and NMDA subunit-specific manner (Liu et al., 1995; Chen and Leonard, 1996; Christie et al., 1999; Liao and Leonard, 1999; Jones and Leonard, 2005) and stimulates NMDA receptor trafficking, and thereby increasing the number of functional NMDA receptors in the cell membrane (Skeberdis et al., 2001).

Recently, insulin-like growth factor-1 has been shown to protect neurons against excitotoxicity caused by kainic acid (Chen et al., 2019). However, the role of insulin in excitotoxicity still remains elusive due to conflicting results from studies. It has been reported that insulin increases the vulnerability of rat cortical neurons to the excitotoxic effects of glutamate, and thereby contributing to cell death (Schäfer and Erdö, 1991, 1992). In contrast, another study demonstrated that insulin blocks glutamate-induced neurotoxicity in differentiated human SH-SY5Y neuroblastoma cells, thereby contributing to cell survival (Nampoothiri et al., 2014). The discrepancy among these results may be due to differences in cell types, culture conditions, and the concentration and timing of glutamate and insulin addition to the culture. Moreover, the development of insulin resistance, caused by prolonged insulin exposure, may mask insulin effects, as neuronal insensitivity to insulin following long-lasting hyperinsulinemia has been shown to potentiate the death of rat cortical neurons upon glutamate treatment (Datusalia et al., 2018). Of note, none of the published studies on the insulin effects in glutamate excitotoxicity (Schäfer and Erdö, 1991, 1992; Nampoothiri et al., 2014; Datusalia et al., 2018) evaluated any parameter of excitotoxicity other than cell survival.

In the present study, we aimed to investigate the effects of insulin on key components of glutamate excitotoxicity in rat cultured cortical neurons, with a focus on cytoplasmic calcium dynamics, DCD, and mitochondrial functioning in single neurons, to clarify the role of insulin signaling in cell survival during glutamate excitotoxicity.

## Materials and Methods

### Materials

Cell culture supplies were obtained from Invitrogen (Thermo Fisher Scientific, Waltham, MA, United States). All other reagents were obtained from Invitrogen or Sigma-Aldrich (Merck, St. Louis, MO, United States).

### Primary Culture of Rat Cortical Neurons

Experiments with animals were performed in accordance with the ethical principles and regulatory documents recommended by the European Convention on the Protection of Vertebrate Animals used for experiments (Guide for the Animals and Eighth Edition. 2010), as well as in accordance with the “Good Laboratory Rules practice”, approved by order of the Ministry of Health of the Russian Federation No. 199n of 04/01/2016. Primary cultures of rat brain cortical neurons were prepared from the cortex of one- or two-day old Wistar rats as previously described (Surin et al., 2014a). The rats were anesthetized, decapitated, and the cortex was removed and separated from the meninges. The extracted tissues were washed in a Ca^2+^- and Mg^2+^-free Hanks solution, crushed, and placed in a papain solution for 15 min at 36°C, washed with standard Hanks solution with phenol red and Minimal Essential Medium (MEM) culture medium, and dispersed in fresh MEM. A homogeneous suspension was precipitated two times in a centrifuge at 200 g for 5 min. The precipitated cells were resuspended to a concentration of 10^6^ cells/ml in neurobasal medium (NBM), supplemented with B-27 Supplement, GlutaMAX, and penicillin/streptomycin. The suspension (200 μl) was transferred onto coverslips attached to the wells of 35 mm plastic Petri dishes (MatTek, Ashland, MA, United States) or a volume of 400 μl into each well of 24-well plastic plates (Corning costar). The glass dishes and plates were pre-coated with 10 mg/ml of polyethyleneimine for 30 min. After one hour, 1.5 ml of NBM, containing 2% B-27 Supplement, 1% antibiotic-antimycotic, and 1% GlutaMAX, was added. The cells were kept in an incubator at 37°C, 95% air + 5% CO_2_, and a relative humidity of 100%. Cytosine arabinoside (AraC, 5 μM) was added to the medium for two or three days to prevent the proliferation of glial cells and obtain cultures with a percentage of neurons of more than 90%. Every three days, the cells were fed by replacing 1/3 of the old medium with new medium. Cultures were used in experiments 10–12 days after plating (10–12 days in culture, DIV). Before every experiment, bottoms with the cells and plates were washed ten times out of the B27 supplement with a buffer containing: (mM): 135 NaCl, 5 KCl, 2 CaCl_2_, 1 MgCl_2_, 20 HEPES, 5 D-glucose; pH 7.4. Then, the cells were kept in this buffer for one hour before every experiment.

### Measurement of [Ca^2+^]_*i*_ and the Mitochondrial Membrane Potential

For ([Ca^2+^]_*i*_) measurements, cortical neurons were loaded with a low affinity Ca^2+^indicator, Fura-FF (2 μM), in the form of acetoxymethyl esters (in AM-form), for 60 min at 37°C to monitor changes of high [Ca^2+^]_*i*_. A non-ionic detergent, PluronicF-127 (0.02%; Molecular Probes, United States), was added to facilitate the penetration of the Fura-FF into the cells. Fura-FF fluorescence was excited alternately at 340 and 380 nm and recorded at 525 nm (dichroic mirror 500 nm). For simultaneous measurements of [Ca^2+^]_*i*_ and the ΔΨ_*m*_, cells were loaded for the last 15 min of the “Fura-FF loading period” in buffer at 37°C with 2.5 μg/ml of Rhodamine 123 (Rh123). Rh123 fluorescence was excited and recorded at 485 and 525 nm, respectively. Accumulation of Rh123 in polarized mitochondria quenches the fluorescent signal. In response to depolarization, the fluorescence is dequenched (Duchen et al., 2003). The measurements were performed at 25–27°Ñ in a medium containing (mM): 135 NaCl, 5 KCl, 2 CaCl_2_, 1 MgCl_2_, 20 HEPES, 5 D-glucose; pH 7.4. Insulin, at 100 nM, was added 5 min prior to 100 μM glutamate, in Mg^2+^ free, 10 μM glycine containing medium. The cells were then exposured to glutamate or glutamate with insulin (100 nM) at 37°C for a period of 15 min. In Ca^2+^-free buffers, CaCl_2_ was replaced with 0.1 mM EGTA and 2 mM MgCl_2_. To examine mitochondrial Ca^2+^ accumulation during glutamate exposure, mitochondria were completely depolarized by adding 1 μM of carbonyl-p- (trifluoromethoxy) phenylhydrazone (FCCP). Finally, 1 μM of Ionomycine (Iono) was added to calibrate the maximal response of Fura-FF to a high rise in [Ca^2+^]_*i*_. Fluorescence measurements were performed using a fluorescence imaging system, which consisted of an Olympus IX-71 inverted microscope equipped with a 175 W xenon lamp, 20 × fluorite objective, a Sutter Lambda 10-2 illumination system (Sutter Instruments, Novato, CA, United States), and a CoolSNAP HQ2 CCD camera operated by the computer program MetaFluor (Molecular Devices, San Jose, CA, United States).

### Measurement of Oxygen Consumption Rates With XF24 Microplate-Based Respirometry

Neuronal oxygen consumption rates (OCR) were measured using the Seahorse XF24 Extracellular Flux Analyzer (Seahorse Bioscience, North Billerica, MA, United States), at 37°C, in a cell medium, consisting of 130 mM NaCl, 5 mM KCl, 2 mM CaCl_2_, 1 mM MgCl_2_, 20 mM HEPES, 5 mM Glucose, and 5 mM NaHCO_3_, at pH ∼ 7.4. The microplate-based respirometry utilizes a 24-well plate format and quantifies the OCR at different times, following the addition of insulin, glutamate, or their vehicles. Prior to each experiment, neurons in each well plate were washed twice with 500 μl of medium. Four wells per plate did not contain neurons, serving as “blank” wells, to control for temperature-sensitive fluctuations in O_2_ fluorophore emission. Following washing, each well was filled with 675 μl of medium and the plates were placed in a CO_2_-free incubator (37°C) for 45 min before each set of measurements to further purge CO_2_ and to allow for temperature and pH equilibration. The plates were then loaded into the XF24 respirometer and further equilibrated for 15 min by 3, 3-min mix, 2-min wait cycles prior to the first measurement. XF24 assays consisted of 3-min mix, 3-min wait, and 2-min measurement cycles and were performed at 37°C. The substances of interest, prepared in assay medium (75 μl), were preloaded into reagent delivery chambers (A–D), and injected sequentially at pre-designated intervals. HEPES (20 mM) was included in medium to ensure pH stability over the two-hour time course of measurements. To evaluate maximum oxygen consumption, carbonyl cyanide p-(trifluoromethoxy) phenylhydrazone (FCCP, 2 μM), rotenone (1 μM), and antimycin A (1 μM) were used. Subsequently, the obtained data was calculated via the Seahorse XF Cell Mito Stress Test Report Generator, which automatically calculates and reports assay parameters. The non-mitochondrial oxygen consumption is the minimum rate measurement after the antimycin A/rotenone injection. The basal respiration is the difference between the last rate measurement before first injection and the non-mitochondrial respiration rate. The maximal respiration is the difference between the maximum rate measurement after FCCP injection and the non-mitochondrial respiration rate. The spare respiratory capacity is the difference between the maximal respiration and the basal respiration.

### ATP Assay

Adenosine triphosphate levels were analyzed in the lysates of cortical neurons using the luciferin-luciferase method. A culture of cortical cells grown in 24-well plates (Corning) for 10–12 DIV was exposed to 100 μM glutamate in the absence or presence of 10–100 nM insulin for 1 h. The cells were then lysed with a 2% trichloroacetic acid/2 mM EDTA solution. The intracellular ATP level was measured using the ATP Assay System Bioluminescence Detection Kit (Promega, Southampton, United Kingdom), using a microplate reader (ClarioStar BMG LABTECH, Germany). The ATP level in each sample was normalized to the protein content (nmol/μg of protein). The Bradford method was used to determine protein concentrations. The average intracellular content of ATP in the control wells of a 24-well plate was taken as 100%. Relative to this, ATP levels in all other samples were calculated.

### Brain-Derived Neurotrophic Factor Assay

Brain-derived neurotrophic factor (BDNF) levels were analyzed in lysates of cortical neurons using a BDNF ELISA Immunoassay (R&D system, McKinley Place, MN, United States). The optical density of the samples was measured on a Picon reader at 450 nm. The level of BDNF in each sample was normalized to the protein content (pg/μg of protein). The Bradford method was used determine protein concentrations. The mean intracellular levels of BDNF in the control wells of the 24-well plate was taken as 100%, and the BDNF levels in all other samples were calculated relative to this.

### MTT Assay

A colorimetric assay based on the reduction of the yellow 3-(4,5-dimethylthiazol-2-yl)-2,5-diphenyl-tetrazolium bromide (MTT) - only in living cells - to dark-blue formazan was used to determine cell viability. Cells were seeded into a 24-well plate (Corning costar 3338, United States). After 10–12 days, cell cultures were exposed for 1 h to glutamate (Glu 100 μM, Mg^2+^-free, 10 μM glycine, and 2 mM Ca^2+^) in the presence or absence of 100 nM insulin. Next, cells were washed with saline (3 × 0.5 ml). NBM was added to the wells, and the cells were returned to the CO_2_ incubator. After 24 h, 20 μl of MTT water solution (4 mg/ml) was added to each well of the 24-well plate. After 30 min, MTT-containing buffer was aspirated, and the cells were dissolved in 300 μl of DMSO. The absorbance of formazan solution was measured at 550 nm using a plate reader (ClarioStar BMG LABTECH, Germany). The optical density of the control group and cell-free wells were considered as 100 and 0% survival, respectively.

### Statistical Analysis

All data are presented as the mean ± standard error of mean (SEM). Statistical analyses were performed with the GraphPad Prism software. For comparing the difference between two groups, the Student’s t-test was used. For comparing the difference between multiple groups, one-way analysis of variance (ANOVA), followed by Tukey’s post-test, comparisons were used. For comparing the difference in dynamics between groups, two-way ANOVA with repeated measures, followed by Tukey’s post-test or Bonferroni post-test, for multiple comparisons was used. Pearson’s correlation coefficient *r* was used to assess the correlations between groups. Statistically significant results are marked with asterisks, ^∗^*p* < 0.05; ^∗∗^*p* < 0.01; ^∗∗∗^*p* < 0.001; and ^∗∗∗∗^*p* < 0.0001.

## Results

### Insulin Protects Cortical Neurons Against Delayed Calcium Deregulation and Mitochondrial Depolarization in Excitotoxicity

**FIGURE 1:**
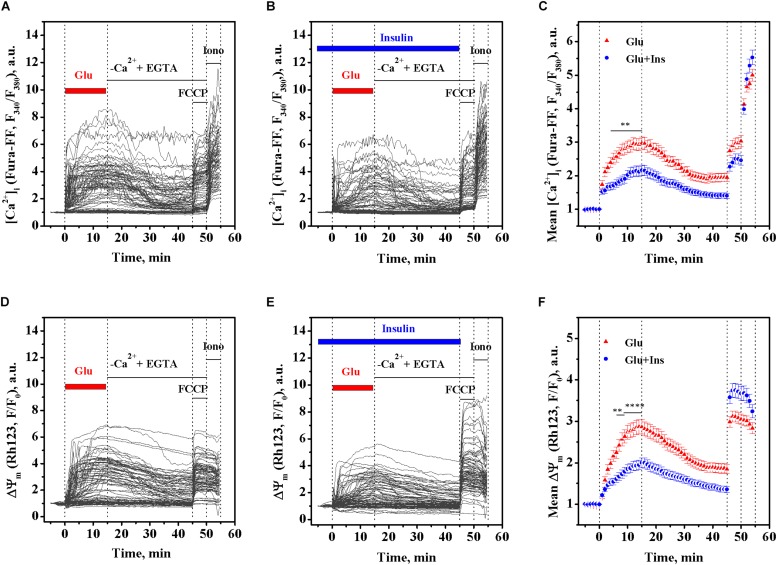
Insulin prevents the rise of intracellular Ca^2+^ ([Ca^2+^]_*i*_) and mitochondrial membrane potential (ΔΨ_*m*_) decrease in excitotoxicity. Fura-FF and Rh123 fluorescence intensities were recorded in cortical neurons exposed to 100 μM glutamate in the presence or absence of 100 nM insulin. **(A)** The dynamics of Fura-FF fluorescence, a measure proportional to [Ca^2+^]_*i*_, in single neurons treated with glutamate in the absence of insulin. **(B)** The dynamics of Fura-FF fluorescence in single neurons treated with glutamate in the presence of insulin. **(C)** The dynamics of Fura-FF fluorescence averaged over groups of neurons treated with glutamate (Glu, *n* = 90) or glutamate with insulin (Glu + Ins, *n* = 90). Data are the mean ± SEM of the number of neurons, *n*. ^∗∗^*p* < 0.01 compared to Glu (two-way ANOVA with repeated measures followed by Bonferroni’s post-test). **(D)** The dynamics of Rh123 fluorescence, a measure inversely proportional to ΔΨ_*m*_, in single neurons treated with glutamate in the absence of insulin. **(E)** The dynamics of Rh123 fluorescence in single neurons treated with glutamate in presence of insulin. **(F)** The dynamics of Rh123 fluorescence averaged over groups of neurons treated with glutamate (Glu, *n* = 87) or glutamate with insulin (Glu + Ins, *n* = 87). Data are the mean ± SEM of the number of neurons, *n*. ^∗∗^*p* < 0.01 and ^∗∗∗∗^*p* < 0.0001 compared to Glu (two-way ANOVA with repeated measures followed by Bonferroni’s post-test).

Primary cultured rat cortical neurons were loaded with a low affinity fluorescent Ca^2+^ indicator Fura-FF and Rh123 dye to simultaneously follow changes in intracellular [Ca^2+^]_*i*_ and the ΔΨ_*m*_, respectively, at 30-s intervals. As expected, the addition of 100 μM glutamate to cultured neurons caused a rapid rise in Fura-FF fluorescence proportional to the rise of [Ca^2+^]_*i*_ (Figure 1A). Along with this, the glutamate treatment resulted in the simultaneous rise of Rh123 fluorescence reciprocal to ΔΨ_*m*_, indicating a ΔΨ_*m*_ decrease (Figure 1D). To examine whether insulin can exert an effect on glutamate-evoked changes of [Ca^2+^]_*i*_ and ΔΨ_*m*_, cultured neurons were pre-treated with 100 nM insulin for 5 min and then exposed to 100 μM glutamate and 100 of nM insulin for the next 15 min (Figures 1B,E). Two-way ANOVA with repeated measures revealed significant effects of time and insulin on both the glutamate-evoked rise of [Ca^2+^]_*i*_ (Figure 1C; *F*_119,20825_ = 259.30, *p* < 0.0001 and *F*_1,175_ = 11.10, *p* = 0.0011, respectively) and mitochondrial depolarization (Figure 1F; *F*_119,20825_ = 158.20, *p* < 0.0001 and *F*_1,175_ = 8.62, *p* = 0.0038, respectively). Bonferroni’s post-test showed that insulin significantly diminished the rise of [Ca^2+^]_*i*_ (*p* < 0.01) and protected against ΔΨ_*m*_ decrease (*p* < 0.01 to *p* < 0.0001) compared to respective non-insulin treated controls, in the period from 4 to 15 min of glutamate stimulation, but not within the first 3 min of glutamate stimulation (*p* > 0.05). At 15 min of glutamate exposure, the mean [Ca^2+^]_*i*_ was decreased by 27.2% (*p* = 0.0017) and the mean ΔΨ_*m*_ increased by 30.2% (*p* < 0.0001) in the insulin treated neurons compared to respective non-insulin treated controls.

**FIGURE 2:**
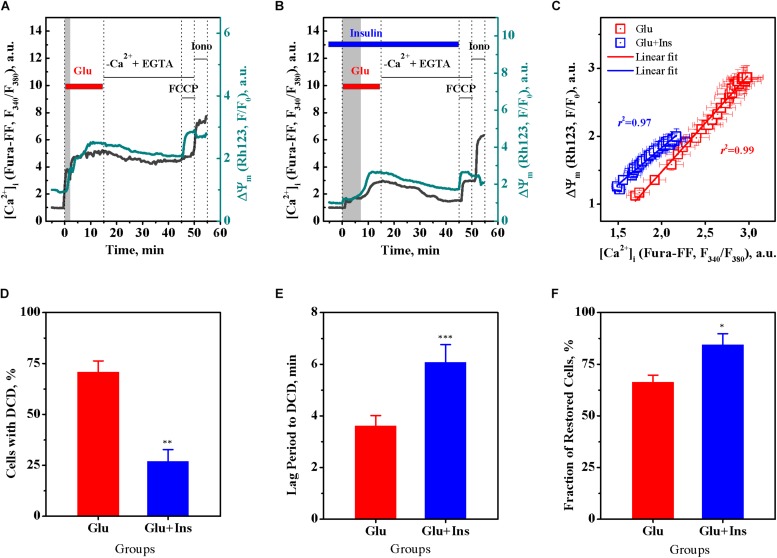
Insulin prevents the onset of delayed calcium deregulation (DCD) in cortical neurons exposed to glutamate. **(A)** Representative dynamics of intracellular Ca^2+^ ([Ca^2+^]_*i*_) and mitochondrial membrane potential (ΔΨ_*m*_) in a single cortical neuron undergoing DCD. **(B)** Representative dynamics of [Ca^2+^]_*i*_ and ΔΨ_*m*_ in a single cortical neuron showing RCE. **(C)** Pearson’s correlation between the mean values of [Ca^2+^]_*i*_ and ΔΨ_*m*_ of Figures 1C,F. The data are the mean ± SEM obtained at every time point, at 30-s intervals, within the 15-min period of glutamate exposure. **(D)** The percentage of neurons undergoing DCD upon treatment with 100 μM glutamate in the absence (Glu) or presence of 100 nM insulin (Glu + Ins). Data are the mean ± SEM of four independent experiments (110 neurons per experiment). ^∗∗^*p* < 0.01 compared to Glu (unpaired two-tailed Student *t*-test). **(E)** The lag period before DCD onset, min. Data are the mean ± SEM of four independent experiments (110 neurons per experiment). ^∗∗∗^*p* < 0.001 compared to Glu (unpaired two-tailed Student *t*-test). **(F)** The percentage of cells restored after RCE in Ca^2+^-free EGTA-containing medium. Data are the mean ± SEM of four independent experiments (110 neurons per experiment). ^∗^*p* < 0.05 compared to Glu (unpaired two-tailed Student *t*-test).

Based on the above data (Figures 1C,F), we performed a correlation analysis using Pearson’s *r*, for the relationship between the [Ca^2+^]_*i*_ and ΔΨ_*m*_ means at every time point, in 30-s intervals, within a period of 15 min of glutamate exposure in the absence or presence of insulin. Figure 2C shows that, during exposure to the same dose of glutamate, the change in the [Ca^2+^]_*i*_ and ΔΨ_*m*_ ranges decreased approximately 1.5 times in presence of insulin. There was a significant linear correlation between [Ca^2+^]_*i*_ and ΔΨ_*m*_ for neurons treated with glutamate in the absence of insulin (Figure 2C; *r* = 0.996, 95% confidence interval 0.991–0.9981; slope 1.43 ± 0.02; *F* = 3374.7, *p* < 0.0001) or in the presence of insulin (*r* = 0.986, 95% confidence interval 0.971–0.994; slope 1.09 ± 0.03; *F* = 1008.4, *p* < 0.0001). Such strong correlations between [Ca^2+^]_*i*_ and ΔΨ_*m*_ values observed at every time point during the entire period of glutamate exposure gives evidence for complete synchrony of [Ca^2+^]_*i*_ and ΔΨ_*m*_ changes in neurons during glutamate action. A comparison of slopes in the Pearson’s equations showed that the observed value for the declined ΔΨ_*m*_, at the same increment of the [Ca^2+^]_*i*_ rise, was on average 24% less in the insulin-treated compared to non-insulin-treated neurons. These results suggest that there is a strong linear relationship between [Ca^2+^]_*i*_ and ΔΨ_*m*_ during glutamate exposure, and although insulin does not change the linearity, it affects the magnitude of the glutamate-induced rise of [Ca^2+^]_*i*_ and ΔΨ_*m*_ decrease.

Figures 2A,B show representative [Ca^2+^]_*i*_ and ΔΨ_*m*_ time curves for single neurons with irreversible DCD and with RCE, respectively. A single neuron undergoing DCD (Figure 2A) upon glutamate exposure has a shorter lag period to DCD, and an irreversible secondary rise of [Ca^2+^]_*i*_ and ΔΨ_*m*_ decrease that cannot be restored upon glutamate removal in Ca^2+^-free EGTA-containing medium, in contrast to a neuron with RCE (Figure 2B). A comparison of groups of neurons demonstrated that insulin significantly decreased the fraction of neurons with DCD (Figure 2D; unpaired two-tailed Student *t*-test; *p* < 0.01), increased the lag period to DCD (Figure 2E; unpaired two-tailed Student *t*-test; *p* < 0.001), and increased the fraction of cells restored after RCE in Ca^2+^-free EGTA-containing medium (Figure 2F; unpaired two-tailed Student *t*-test; *p* < 0.05), as compared to non-insulin treated controls. Together, these results suggest that insulin protects cultured cortical neurons against glutamate excitotoxicity by preventing the DCD onset and development.

### MK 801 Abrogates the Effects of Insulin and Glutamate on [Ca^2+^]_*i*_ and ΔΨ_*m*_

**FIGURE 3:**
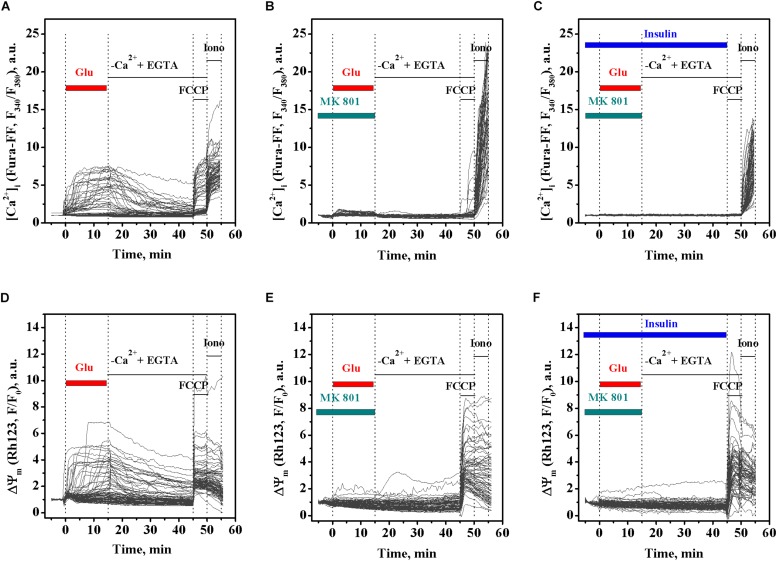
MK 801 inhibits the glutamate-evoked changes in intracellular Ca^2+^ ([Ca^2+^]_*i*_) and mitochondrial membrane potential (ΔΨ_*m*_) in cortical neurons in the presence and absence of insulin. **(A)** The dynamics of Fura-FF fluorescence, the measure proportional to [Ca^2+^]_*i*_, in single neurons exposed to glutamate. **(B)** The dynamics of Fura-FF fluorescence in single neurons exposed to glutamate in the presence of 10 μM MK 801. **(C)** The dynamics of Fura-FF fluorescence in single neurons exposed to insulin and glutamate in the presence of 10 μM MK 801. **(D)** The dynamics of Rh123 fluorescence, the measure inversely proportional to ΔΨ_*m*_, in single neurons exposed to glutamate. **(E)** The dynamics of Rh123 fluorescence in single neurons exposed to glutamate in the presence of 10 μM MK 801. **(F)** The dynamics of Rh123 fluorescence in single neurons exposed to insulin and glutamate in the presence of 10 μM MK 801.

MK 801 is an inhibitor of Ca^2+^ influx, which physically blocks ion permeation through the NMDA receptor ion channel upon binding to the ion channel vestibule (Song et al., 2018). MK 801 has been shown to completely abrogate DCD development in excitotoxicity by blocking Ca^2+^ influx through the NMDA receptor and the plasmalemmal Na^+^/Ca^2+^ exchanger operating in reverse mode (NCX_*rev*_) (Kiedrowski and Costa, 1995; Brittain et al., 2012). To examine whether the insulin effects on the glutamate-evoked rise of [Ca^2+^]_*i*_ and the ΔΨ_*m*_ decrease relate to the Ca^2+^ influx, we investigated the effects of MK 801 on the dynamics of [Ca^2+^]_*i*_ and ΔΨ_*m*_ in cultured rat cortical neurons upon glutamate exposure in the presence or absence of insulin. As expected, treatment with 100 μM glutamate resulted in the rise of [Ca^2+^]_*i*_ (Figure 3A), simultaneously with the drop in the ΔΨ_*m*_ (Figure 3D). Two-way ANOVA with repeated measures revealed significant effects of time and treatment on the glutamate-evoked rise of [Ca^2+^]_*i*_ (Figures 3A–C; *F*_118,25960_ = 792.45, *p* < 0.0001 and *F*_2,220_ = 20.43, *p* < 0.0001, respectively) and interaction (*F*_236,25960_ = 54.76, *p* < 0.0001). Tukey’s post-test showed that MK 801 significantly and almost completely prevented the rise of [Ca^2+^]_*i*_ (Figure 3B; *p* < 0.05 to *p* < 0.0001) compared to the time-matched controls (Figure 3A). Two-way ANOVA with repeated measures, followed by Tukey’s post-test, showed that MK 801 also prevented the glutamate-induced ΔΨ_*m*_ decrease (Figure 3E; *p* < 0.001 to *p* < 0.0001) compared to the time-matched non-MK 801 treated controls (Figure 3D). There was no significant difference in [Ca^2+^]_*i*_ and ΔΨ_*m*_ dynamics between neurons exposed to glutamate and insulin (Figures 3C,F, respectively) or glutamate alone (Figures 3B,E, respectively) in the presence of MK 801 within the entire 15-min period of glutamate exposure (two-way ANOVA with repeated measures followed by Tukey’s post-test; *p* > 0.05). These results suggest that the insulin effects on glutamate-induced the [Ca^2+^]_*i*_ and ΔΨ_*m*_ dynamics relate to the Ca^2+^ influx.

### Insulin Improves the Viability of Neurons Exposed to Glutamate

**FIGURE 4:**
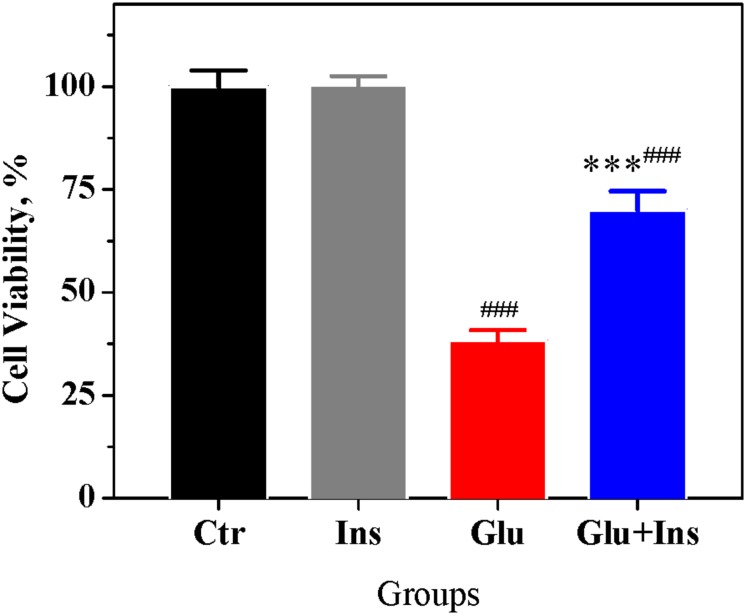
Insulin improves the viability of cortical neurons in excitotoxicity. The percentage of viable cells after treatment with 100 nM insulin (Ins), 100 μM glutamate (Glu), a combination of glutamate and insulin (Glu + Ins), or no treatment (Ctr). Data are the mean ± SEM of six independent experiments. ^###^*p* < 0.001 compared to Ctr; ^∗∗∗^*p* < 0.01 compared to Glu (one-way ANOVA followed by Tukey’s post-test for multiple comparisons).

Given that DCD is considered to be the point-of-no-return in excitotoxicity (Nicholls, 2004), using the MTT assay, we investigated whether insulin can improve the viability of primary cultured cortical neurons exposed to 100 μM glutamate. Primary cultured cortical neurons were treated with glutamate in the presence or absence of 100 nM insulin for 1 h, and after 24 h, cell viability was assayed. One-way ANOVA demonstrated a significant difference in cell viability between groups (Figure 4; *F*_3,20_ = 72.53, *p* < 0.0001). Tukey’s post-test showed that glutamate decreased the viability of cells by 30% (*p* < 0.001) and 61% (*p* < 0.001) in the presence and absence of insulin, respectively, as compared to the control. Insulin significantly improved the viability of neurons exposed to glutamate (*p* < 0.001) compared to neurons exposed to glutamate alone. Together, these results suggest that insulin improves the viability of cortical neurons in glutamate excitotoxicity.

### Insulin Improves Oxygen Consumption Rates in Excitotoxicity

**FIGURE 5:**
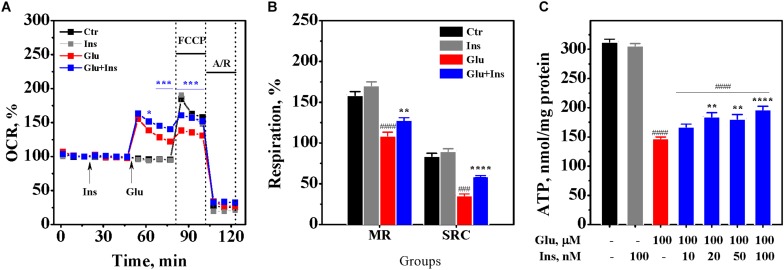
Insulin improves mitochondrial oxidative metabolism and prevents adenosine triphosphate (ATP) depletion in excitotoxicity. **(A)** Oxygen consumption rates (OCR) in cortical neurons exposed to 100 μM glutamate (Glu), 100 nM insulin (Ins), 100 nM insulin and 100 μM glutamate (Glu + Ins), or no treatment (Ctr). A/R, antimycin A/rotenone. Data are representative of four independent experiments (*n* = 6–14 wells, mean ± SEM). ^∗^*p* < 0.05, ^∗∗^*p* < 0.01 and ^∗∗∗^*p* < 0.001 compared to Glu (two-way ANOVA with repeated measures followed by Tukey’s post-test). **(B)** Maximal respiration (MR) and spare respiratory capacity (SRC) in neurons exposed to 100 μM glutamate (Glu), 100 nM insulin (Ins), 100 nM insulin and 100 μM glutamate (Glu + Ins), or no treatment (Ctr). Data are representative of four independent experiments (*n* = 6–14 wells, mean ± SEM). ^####^*p* < 0.0001 compared to Ctr; ^∗∗^*p* < 0.01 and ^∗∗∗∗^*p* < 0.0001 compared to Glu (one-way ANOVA followed by Tukey’s post-test). **(C)** ATP levels in lysates of cortical neurons exposed to glutamate (Glu) and insulin (Ins) at the indicated concentrations. Data are the mean ± SEM of four independent experiments. ^####^*p* < 0.0001 compared to non-treated control; ^∗∗^*p* < 0.01 and ^∗∗∗∗^*p* < 0.0001 compared to neurons exposed to 100 μM glutamate (one-way ANOVA followed by Tukey’s post-test comparisons).

To examine whether insulin can exert an effect on neuronal respiration during excitotoxicity, we measured OCR, a reliable readout of mitochondrial activity, in cultured neurons pre-treated with 100 nM insulin and then exposed to 100 μM glutamate and 100 nM insulin or neurons treated with 100 μM glutamate alone, 100 nM insulin alone, or no treatment (control) (Figure 5A). Two-way ANOVA with repeated measures revealed significant effects of time and treatment on the OCR (*F*_16,464_ = 1249.77, *p* < 0.0001 and *F*_3,29_ = 13.16, *p* < 0.0001, respectively) and interaction (*F*_48,464_ = 43.34, *p* < 0.0001). Tukey’s post-test for multiple comparisons showed that glutamate, as expected, significantly increased the OCR (*p* < 0.001 to *p* < 0.0001) compared to the control. Insulin significantly increased the OCR by on average of 16% in neurons exposed to glutamate (*p* < 0.05 to *p* < 0.001) compared to neurons exposured to glutamate alone, while there was no difference between neurons exposed to insulin alone and the control neurons (*p* > 0.05).

To assess the effects of insulin and glutamate exposure on the maximal respiration and the spare respiratory capacity, we measured the OCR upon addition of FCCP, which raises the OCR to a maximal value, followed by addition of antimycin A with rotenone that reduces the OCR to a minimal value. One-way ANOVA demonstrated a significant difference in the maximal respiration between groups (Figure 5B; *F*_3,29_ = 40.17, *p* < 0.0001), but not in the basal respiration (*F*_3,29_ = 1.73, *p* = 0.18). There was a significant difference between groups in the spare respiratory capacity (*F*_3,29_ = 97.52, *p* < 0.0001). Tukey’s post-test for multiple comparisons showed that glutamate significantly decreased the maximal respiration by 31% (*p* < 0.0001) and the spare respiratory capacity by 58% (*p* < 0.0001) compared to the control. Insulin significantly improved both the maximal respiration and the spare respiratory capacity in the glutamate treated neurons (Figure 5B; *p* = 0.007 and *p* < 0.0001, respectively). These results suggest that insulin improves mitochondrial oxidative metabolism during excitotoxicity.

### Insulin Prevents Glutamate-Induced ATP Depletion

To examine whether insulin can prevent glutamate-induced ATP depletion, we measured ATP levels in the lysates of cultured rat cortical neurons exposed to 100 μM glutamate in the absence or presence of insulin ranging from 10 to 100 nM. One-way ANOVA showed significant differences between groups (Figure 5C; *F*_6,21_ = 136.4, *p* < 0.0001). Tukey’s post-test revealed that glutamate evoked a significant depletion of ATP levels by 53% (*p* < 0.0001) compared to the non-treated control. Insulin significantly and dose-dependently restored ATP levels in the glutamate treated neurons, with the highest insulin dose increasing ATP levels by 33% (Figure 5C; *p* < 0.0001). This result suggests that insulin prevents the depletion of ATP levels in neurons during excitotoxicity.

### Insulin Prevents the Decrease of BDNF Levels in Excitotoxicity

**FIGURE 6:**
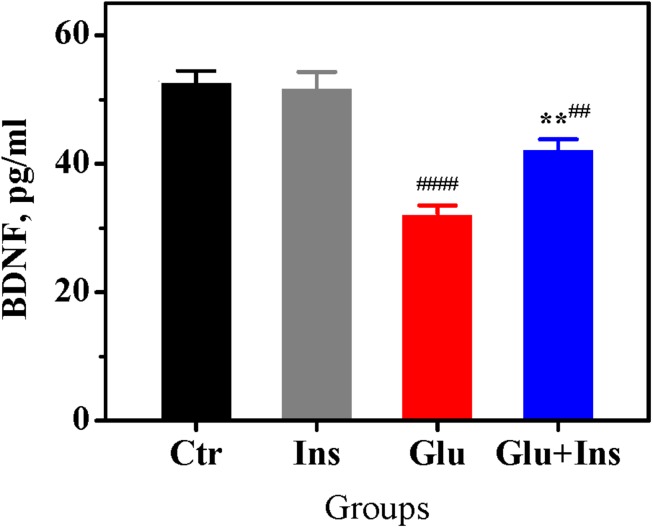
Insulin prevents decrease of brain-derived neurotrophic factor (BDNF) levels in rat cortical neurons exposed to glutamate. BDNF levels in the untreated neurons (Ctr) and neurons treated with 100 μM glutamate (Glu), 100 nM insulin (Ins), or 100 μM glutamate with 100 nM insulin (Glu + Ins). Data are the mean ± SEM of six independent experiments. ^##^*p* < 0.01, ^####^*p* < 0.0001 compared to Ctr; ^∗∗^*p* < 0.01 compared to Glu (one-way ANOVA followed by Tukey’s post-test).

Prolonged exposure to glutamate has been shown to cause a significant decrease in BDNF levels in neurons, accompanied with reduced cell viability and enhanced cell apoptosis (Wang et al., 2008). To examine whether insulin can affect the glutamate-induced decrease in neuronal BDNF levels, we measured BDNF levels in lysates of cultured rat cortical neurons exposed to 100 μM glutamate in presence or absence of 100 nM insulin. One-way ANOVA revealed a significant difference between groups (Figure 6; *F*_3,20_ = 28.95, *p* < 0.0001). Tukey’s post-test showed that glutamate induced a significant decrease in BDNF levels by 39% (*p* < 0.0001) compared to the control, while insulin significantly ameliorated the effect of glutamate (*p* = 0.0038).

## Discussion

The main finding of our study is that insulin protects cortical neurons against excitotoxicity by affecting the glutamate-evoked rise of [Ca^2+^]_*i*_ and preventing the onset of DCD. Prolonged glutamate exposure causes a typical biphasic cytosolic Ca^2+^ response in neurons, with a fast initial [Ca^2+^]_*i*_ increase, followed—after a lag period—by a larger secondary [Ca^2+^]_*i*_ rise, simultaneous to a collapse of the ΔΨ_*m*_ (Tymianski et al., 1993; Nicholls and Budd, 1998, 2000; Vergun et al., 1999; Khodorov, 2004). The secondary rise of [Ca^2+^]_*i*_, when irreversible, is postulated to be the point-of-no-return in excitotoxicity and is referred to as “delayed calcium deregulation” (Nicholls and Budd, 2000; Nicholls, 2004; Gerencser et al., 2009). DCD occurs when a massive Ca^2+^ influx (predominantly via NMDA receptors) is no longer counterbalanced by a Ca^2+^ efflux, which is due to the plasmalemmal Na^+^/Ca^2+^ exchanger switching to the reverse operating mode (NCX_*rev*_), and thereby redirecting the flow of Ca^2+^ into the cell (Kiedrowski and Costa, 1995; Brittain et al., 2012). Events that occur downstream of DCD onset are considered to influence the timing of cell death without altering its inevitability. In this study, we demonstrated for the first time that insulin significantly diminished the glutamate-evoked rise of [Ca^2+^]_*i*_ and drop of ΔΨ_*m*_ (Figure 1), decreased the fraction of neurons undergoing DCD (Figure 2D), increased the lag period to DCD (Figure 2E), and increased the fraction of cells restored after RCE in Ca^2+^-free EGTA-containing medium (Figure 2F). Collectively, these results suggest that insulin prevents DCD onset and development, and therefore predictably influences the viability of cells in excitotoxicity. In line with these results, using the MTT assay, we demonstrated that insulin improves the viability of cultured cortical neurons during prolonged glutamate exposure (Figure 4).

The glutamate-evoked rise of [Ca^2+^]_*i*_ and drop of ΔΨ_*m*_, as well as the development of DCD, in our experiments, was completely inhibited by MK 801 (Figure 3), the inhibitor of Ca^2+^ influx both the NMDA ion channel and the plasmalemmal NCX_*rev*_ (Brittain et al., 2012), indicating that the insulin effects on DCD onset relates to its action on the Ca^2+^ influx. Given that insulin has been reported to potentiate the NMDA receptor-mediated current (Liu et al., 1995; Chen and Leonard, 1996; Christie et al., 1999; Liao and Leonard, 1999; Skeberdis et al., 2001; Jones and Leonard, 2005), we speculate that NCX_*rev*_ may be a target candidate of insulin action in excitotoxicity, presumably because of insulin’s effect on cell metabolism.

A synchrony of DCD and mitochondrial depolarization in neurons exposed to glutamate has been previously demonstrated (Khodorov, 2004; Abramov and Duchen, 2008; Surin et al., 2014b). Herein, we found for the first time that insulin prevented both the glutamate-induced [Ca^2+^]_*i*_ rise (Figures 1A–C) and the decrease in ΔΨ_*m*_ (Figures 1D–F). The strong linear Pearson’s correlation between the [Ca^2+^]_*i*_ and the ΔΨ_*m*_ values, at every time point during glutamate exposure, provides evidence for the complete synchrony of [Ca^2+^]_*i*_ and ΔΨ_*m*_ (Figure 2C). Insulin did not change the linearity of the relationship between [Ca^2+^]_*i*_ and ΔΨ_*m*_, but diminished magnitude of the [Ca^2+^]_*i*_ rise and ΔΨ_*m*_ decrease in excitotoxicity. A comparison of slopes in the Pearson’s equations showed that the ΔΨ_*m*_ decline, at the same increment the [Ca^2+^]_*i*_ increased, was on average 24% less in the insulin treated neurons compared to the non-insulin treated neurons. This result seems to indicate that in addition to its effect on the calcium-dependent ΔΨ_*m*_ decrease, insulin preserves the ΔΨ_*m*_ in a calcium-independent way, presumably through its known stimulatory action on mitochondrial metabolism. For the reference, it has been shown that insulin specifically activates the mitochondrial tricarboxylic acid cycle (TCA), increasing the oxidation of ^14^C labeled pyruvate to CO_2_ by 30% (Bessman and Mohan, 1997).

We found for the first time that insulin significantly improved the OCR (Figure 5A), the maximal respiration, and the spare respiratory capacity (Figure 5B) in neurons exposed to glutamate. Moreover, insulin ameliorated ATP depletion induced by glutamate in a dose-dependent manner (Figure 5C). As reviewed by Rueda et al. (2016), activation of the NMDA receptor results in a Ca^2+^-dependent transient stimulation of respiration, which drops rapidly in the continuous presence of glutamate or NMDA. In view of growing evidence, this insulin effect can be linked to the activation of poly(ADP-ribose) polymerase-1 (PARP-1). The high PARP-1 activity is causal to the drop of respiration and ATP depletion under prolonged NMDA exposure (Rueda et al., 2015). PARP-1 blocks glycolysis through the depletion of the cytosolic NAD^+^ pool and direct inhibition of hexokinase-1, a key glycolytic enzyme (Fouquerel et al., 2014). In line with this, pyruvate addition preserves OCR from impairments caused by glutamate (Clerc et al., 2013; Laird et al., 2013). Although the exact mechanisms of PARP-1 activation during excitotoxicity remain unclear, evidence suggests that there is a relationship between PARP-1 hyperactivation and intracellular Ca^2+^ under this condition (Abramov and Duchen, 2008; Sorokina et al., 2018). Ca^2+^ chelation has been shown to abrogate PARP-1 hyperactivation induced by peroxynitrite (Virág̀ et al., 1999) and hydrogen peroxide (Bentle et al., 2006), indicating that Ca^2+^ is at least an important co-factor in PARP-1 hyperactivation after ROS-induced DNA damage. In this context, the effects of insulin on OCR (Figures 5A,B) and ATP levels (Figure 5C) in excitotoxicity seem to be secondary to its preventive effect on the glutamate-induced elevation of [Ca^2+^]_*i*_ and DCD onset.

There is evidence that the BDNF deficiency is a factor contributing to glutamate excitotoxicity. BDNF, a member of the neurotrophin family, has been shown to protect neurons against glutamate-induced cell death via the activation of several signaling pathways (Hashimoto et al., 2002; Almeida et al., 2005), while a deficit in BDNF leads to reduced cell viability and enhanced cell apoptosis in excitotoxicity (Wang et al., 2008). In the present study, we demonstrated for the first time that insulin significantly ameliorates the glutamate-induced decrease in BDNF levels (Figure 6). Thus, insulin may preserve the neuroprotective BDNF signaling in excitotoxicity.

In this study we examined the effects of short-term insulin exposure (20–60 min) on glutamate excitotoxicity in cultured cortical neurons, unlike previous *in vitro* studies demonstrating effects of 18- and 24-hour chronic insulin treatment (Schäfer and Erdö, 1991, 1992). Prolonged insulin exposure for 24 hours has been recently shown to induce insulin insensitivity and potentiate the death of rat cortical neurons upon glutamate exposition (Datusalia et al., 2018). The findings of Datusalia et al. (2018) could explain why chronic insulin treatment increased the vulnerability of rat cortical neurons to the excitotoxic effects of glutamate (Schäfer and Erdö, 1991, 1992), in contrast to our study, which showed the protective effects *in vitro* of a short-term insulin treatment. The maximum activation of insulin receptors in neurons has been observed as early as 5 to 15 min following insulin stimulation (Persiyantseva et al., 2013). Therefore, the short-term exposure to insulin is sufficient to produce an effect. The 20- and 60-min insulin exposure period in our experiments seems to be relevant to *in vivo* conditions of intranasal insulin therapy. The intranasally administered insulin has been shown to reach a peak value in the rat brain regions at 15 min, and then decline gradually overtime by about 2-fold at 60 min (Fan et al., 2019). The effects of short-term insulin exposure observed in our study does not contradict the results of intranasal insulin therapy, which has already been shown to be effective in the treatment of Alzheimer’s disease (Craft et al., 2012) and experimental traumatic brain injury (Brabazon et al., 2017), for which glutamate excitotoxicty is an accompanying condition or symptom.

## Conclusion

In conclusion, we found that the short-term exposure to insulin protected cultured cortical neurons against excitotoxicity in at least two ways: (a) by affecting the glutamate-evoked rise of intracellular calcium and preventing the onset of delayed calcium deregulation, the point-of-no-return in excitotoxicity; (b) by improving oxygen consumption rates, oxidative mitochondrial metabolism, and preserving the decrease in intracellular ATP levels. Insulin ameliorated the glutamate-induced production mitochondrial depolarization, decreased ATP production, and diminished levels of the neurotrophin BDNF, which is a critical neuroprotector in excitotoxicity. In contrast to chronic insulin exposure that induces neuronal insulin resistance, the adverse factor in excitotoxicity, the short-term insulin exposure in our experiments was shown to be a protective treatment against excitotoxicity.

## Data Availability Statement

Datasets are available on request. The raw data supporting the conclusions of this manuscript will be made available by the authors, without undue reservation, to any qualified researcher.

## ETHICS STATEMENT

The animal study was reviewed and approved by the ethics committee of National Medical Research Center for Children’s Health, Ministry of Health of Russian Federation.

## Author Contributions

VP, IK, AF, and IP conceived and designed the experiments. IK, MB, DB, and AD performed the experiments. IK and IP analyzed the data. IK, AS, and VP contributed with the Materials and Methods, and critically revised the manuscript. ES received data on changes in the content of ATP and BDNF. All authors wrote and approved the final version of the manuscript.

## Conflict of Interest

The authors declare that the research was conducted in the absence of any commercial or financial relationships that could be construed as a potential conflict of interest.
